# Willingness to use PrEP among female university students in Lesotho

**DOI:** 10.1371/journal.pone.0230565

**Published:** 2020-03-31

**Authors:** Dimitris Karletsos, Charlotte R. Greenbaum, Emily Kobayashi, Margaret McConnell

**Affiliations:** 1 Tulane University School of Public Health and Tropical Medicine, New Orleans, Louisiana, United States of America; 2 Harvard T.H. Chan School of Public Health, Boston, Massachusetts, United States of America; 3 Clinton Health Access Initiative, Boston, Massachusetts, United States of America; Ohio State University, UNITED STATES

## Abstract

Oral pre-exposure prophylaxis (PrEP) for HIV-negative individuals at high risk was introduced in Lesotho in April 2016. To assess the feasibility and acceptability of PrEP in Lesotho and to study the attitudes and beliefs around HIV risk and prevention measures among young women, between September and December 2016 we asked 302 female university students at fourteen higher education institutions in Lesotho about their sexual behavior, experiences of sexual coercion and abuse, HIV risk perception, willingness to use PrEP, as well as their attitudes toward condom use and self-administration of daily medications. Overall, 57.3% of the sample reported perceiving themselves at risk of acquiring HIV and 32.1% reported being strongly willing to use PrEP if it were available in their community. In a multivariate mediation analysis, perceived HIV risk was associated with 11.5 percentage points increase in likelihood of using PrEP (p = 0.041). Multiple concurrent sexual partnership was associated with 16.1 percentage points increase in likelihood of self-perceived HIV risk (p = 0.007), while having sexual partners in polygamous relationships was associated with 17.8 percentage points increase in likelihood of self-perceived HIV risk (p = 0.002) and the mediated indirect effect accounted for 18.2% of its total effect. Those who reported strong adherence to antibiotics were 23.1 percentage points more likely to express willingness to use PrEP than those who did not (p = 0.004), and those who reported to dislike condoms were 19.1 percentage points more likely to be willing to use PrEP than those who did not report aversion to condom use: these effect were direct and not mediated by HIV risk perception. Intimate partner violence (IPV) in the network of peers was also directly associated with willingness to use PrEP and its effect was not significantly mediated by HIV risk perception: those who had friends who experienced intimate partner violence were 14.9 percentage points more likely to be willing to use PrEP than those who did not report IPV in their network of peers (p = 0.009). These findings support the inclusion of individuals with multiple concurrent sexual partners among the key populations for PrEP provision and confirm that willingness to use PrEP is not solely driven by HIV risk perception. They also indicate that the presence of IPV in peer networks is related to one’s willingness to use PrEP. PrEP service provision may generate synergies with IPV prevention programs when offered within this framework.

## Introduction

In April 2016, the Government of Lesotho’s Ministry of Health (MoH) released new guidelines on the use of antiretroviral therapy for HIV prevention and treatment. These guidelines introduced oral pre-exposure prophylaxis (PrEP) as part of the national HIV prevention strategy [1]. PrEP refers to the use of antiretroviral drugs in HIV-negative people to reduce their risk of acquiring HIV infection. A combination of two HIV medicines (tenofovir and emtricitabine), sold under the name Truvada, is approved for daily use as PrEP. Studies have shown that PrEP is highly effective at preventing HIV if it is used as prescribed [2–5]. PrEP alone helps reduce the risk of HIV infection in the absence of other prevention methods, such as condoms. The risk of getting HIV from sex is further reduced if PrEP is used in combination with condoms or other prevention methods; however, PrEP is much less effective when it is not taken consistently on a daily basis [6].

The 2016 MoH guidelines include recommendations for healthcare providers to determine the eligibility of subjects who may benefit from PrEP: a risk assessment should be conducted for HIV-negative individuals and should inquire about known high-risk behaviors that increase one’s likelihood of acquiring HIV. Nevertheless, although an individual risk assessment is always recommended, the MoH guidelines list key populations to whom PrEP services should be particularly targeted. These include: those who have exchanged sex for money or paid for sex; men who have sex with men and transgender individuals; people who inject drugs; people in sero-discordant relationships where the HIV-positive partner is not on ART; those in sero-discordant relationships where the HIV-positive partner has been on ART for less than 12 months; those in sero-discordant relationships where the HIV-positive partner’s viral load is >1,000 copies/ml or recent viral load is not known but partner’s ART adherence is documented to be poor; those with multiple concurrent sexual partners; and incarcerated individuals. Additionally, intimate partner violence (IPV) is identified as one of the main drivers of the HIV epidemic in Lesotho in the 2016 Ministry of Health HIV testing guidelines; however, people who experience IPV are not explicitly mentioned among the key populations to whom PrEP should be targeted.

Across all populations, young women aged 15–29 are among those with the highest estimated HIV incidence in Lesotho, equal to 1.9 new infections per 100 person-years of exposure [7]. Multiple factors have been associated with the increased HIV incidence in this population. Either because they experience gender-based inequality or because they lack economic empowerment, young women may exchange sex for money or economic support, and thus meet the definition of high-risk population to whom PrEP provision should be targeted. In addition to this, young women, being less likely to be married, may have multiple concurrent sexual partners and therefore be eligible for targeted PrEP service provision, as per 2016 MoH guidelines. Furthermore, young women who exchange sex for money or economic support experience gender power imbalance which may translate into other forms of coercion and abuse. The association between transactional sex and intimate partner violence (IPV) has been long established [8,9]: for instance, Wood and Jewkes [8] found that women who engage in transactional sex with men are more likely to experience IPV. Women may feel forced to remain in these relationships if the partner provides critical income. There is a demonstrated association between transactional sex and incidence of HIV infection [10,11]. Power imbalance in sexual interactions has also been previously described as a barrier to condom use negotiation [12]. As for multiple concurrent sexual partnerships, mixed evidence has been reported on the role of the network effect in the HIV epidemic [13–15]. Nevertheless, multiple concurrent sexual partnerships have been associated with an increased risk of HIV infection in endemic areas by several researchers, both in mathematical model studies [16] and comparative studies of sexual behavior [17,18]. This research informed the inclusion of those who have exchanged sex for money and those with multiple concurrent sexual partners in the 2016 guidelines listing key populations to whom PrEP service provision should be targeted. A combination of these factors may affect young women and contribute to the observed increased incidence among this key population; it is therefore important to understand to what extent do these factors translate into actual HIV risk self-perception and willingness to use PrEP.

In particular, female university students may face several of the potential risk factors that affect the broader women’s population, which are included in the MoH HIV testing and PrEP guidelines: they are part of the age and gender group with the highest estimated incidence, they may face gender-based violence and IPV, they may engage in transactional sex because they lack economic stability, and may as well have multiple concurrent sexual partners because, due to their younger age, they are less likely to be married than their older peers. It is of public health interest to understand whether female university students who meet the MoH criteria for PrEP targeting would be willing to use PrEP; it is also important to understand whether there exist other determinants for willingness to use PrEP in this population; finally, because PrEP use may be influenced, among other factors, by one’s HIV risk self-perception, it is important to identify what are the factors that more than others affect women’s HIV risk self-perception and to what extent does this latter translate into interest in using PrEP. Therefore, the purpose of this study is to assess HIV risk perception and willingness to use PrEP, as well as their associations to condom use and negotiation, exposure to IPV, sexual coercion and other risk factors among a subpopulation of young women in Lesotho, representative of those attending tertiary education institutions, in order to inform HIV prevention strategies and future operational research on integrated service provision in Lesotho.

## Methods

### Participants

Between September and December 2016, we asked 401 female university students at fourteen higher education institutions in Lesotho about their sexual behavior, experiences of sexual coercion and abuse, HIV risk perception, willingness to use PrEP, as well as their attitudes toward condom use and self-administration of daily medications. This cross-sectional study included other components such as family planning and attitudes towards contraceptive use, whose results are not discussed in the present manuscript.

Female students from each of the country’s tertiary institutions were sampled using a stratified simple random sampling with proportional allocation across institutions, each representing a stratum. The sample size per institution was proportional to the size of the student body of that institution. A complete list of female students enrolled at each institution was obtained and each element of the list was assigned with a random number using a table of random digits; elements in the list were then sorted in ascending order and the first *n* elements were selected to be part of the survey, where *n* is equal to the sample size. To be eligible, participants had to be enrolled at the time of the survey in one of the higher education institutions listed in Table 1, be between sixteen and twenty-nine years old, female, and able and willing to provide written informed consent in English or Sesotho as approved by the Lesotho Ministry of Health Institutional Review Board. Students were not compensated for completing the paper-based survey, which took about 40 minutes to complete. Out of 401 students approached, 11 refused to participate, 390 completed the survey and 310 students answered whether they perceived themselves at risk of HIV, or whether they had already been diagnosed with HIV. Those who did not answer about their HIV risk self-perception or HIV status were not statistically different from those who answered in terms of age, HIV risk factorsor gender-based violence predictors. Of the 310 participants who answered about their HIV risk self-perception or HIV status, 8 students reported being HIV-positive. We limited the analysis to 302 self-reported HIV-negative female students who disclosed their HIV risk self-perception.

**Table 1:** Analytic sample descriptive statistics.

n = 302	n (%)
University or School:	
Centre for Accounting Studies (CAS)	14 (4.64)
Roma College of Nursing (RCN)	2 (0.66)
Scott’s College of Nursing (SCN)	2 (0.66)
Paray School of Nursing (PSN)	2 (0.66)
Maluti School of Nursing (MSN)	3 (0.99)
Botho University (BU)	3 (0.99)
Lesotho College of Education (LCE)	63 (20.86)
Lesotho Institute of Public Administration and Management (LIPAM)	5 (1.66)
National Health Training College (NHTC)	12 (3.97)
Lesotho Agricultural College (LAC)	6 (1.99)
Limkokwing University of Creative Technology (LUCT)	36 (11.92)
Lerotholi Polytechnic (LP)	20 (6.62)
Institute of Development Management (IDM)	7 (2.32)
National University of Lesotho (NUL)	127 (42.05)
Ever been forced to have sex	118 (39.07)
Have friends who were beaten by male partner	129 (42.71)
Have friends who have had sex for money	85 (28.14)
Find it hard to negotiate condom use	113 (37.42)
Report inconsistent condom use	102 (33.77)
Report to dislike condoms	50 (16.55)
Report strong adherence to antibiotics	42 (13.91)
More than one current sexual partner	118 (39.07)
Partner has multiple partners or unsure	179 (59.27)
Had STIs in past 12 months or unsure	67 (22.18)
Age	μ (SD)
	21.82 (2.98)

### Measures

Data on demographic characteristics, HIV risk factorsand risk perception, condom use and condom negotiation, gender-based violence indicators, and willingness to use PrEP were collected using self-administered questionnaires.

Peer networks have been reported to influence men’s perpetration of IPV through the internalization of peer network norms, the pressure to conform to peer network norms and the direct involvement of peers in shaping couple power dynamics [19]. Indirect questioning to estimate intimate partner violence in population surveys has been validated by previous studies [20,21]. Therefore, to deal with the information bias that may be generated by self-reporting on intimate partner violence, we used intimate partner violence in the respondent’s network of peers as a proxy to estimate women’s experience of intimate partner violence. We used the question “Were any of your friends hit or beaten by their boyfriends or husbands?” and coded the answers in a binary “yes” or “no” variable with a third choice to capture “don’t know” answers.

In addition to IPV, sexual exploitation and abuse is another form of gender-based violence. To capture this information we asked participants whether they had ever had sex for money and whether, to their knowledge, any of their peers had: responses were captured as binary variables taking the values of “yes” or “no”, with an additional option coding “don’t know” answers. Furthermore, we asked participants whether they had ever been forced to have sex: answers to the question “How many times have you been forced or pushed to have sex against your will?” were recoded into a binary variable taking a value of 1 (“yes”) in the presence of any occurrence and 0 (“no”) otherwise. To elicit information about respondents’ sexual networks characteristics we asked whether they currently had a sexual partner (“Are you in a relationship at the moment?”) and whether they currently had one or multiple partners (“How many partners do you currently have?”); furthermore, we asked whether the respondents’ main partner was in an monogamous relationship with the respondent or not (“Does your husband or main partner have other partners that you know of?”), based on the fact that customary law allows polygamy in Lesotho.

Imperfect condom use was defined using the question “In the last 6 months, how often did you use condoms when you had sex?” and was encoded with a value of 0 (“no”) if the selected answer was “always” and with a value of 1 (“yes”) if the answer was any among the prompted options “most of the times”, “sometimes”, “rarely” or “never”. Negative attitude towards condom use was captured by asking the following question “To what extent do you agree with the following statement? ‘I do not like condoms’” and recoding the answer as 1 if the respondent answered “agree” or “strongly agree”. Additionally, information on condom negotiation was elicited by asking “In your experience, how hard is it to negotiate condom use with men?” and recoded as 1 (hard condom negotiation) if the respondent answered “hard” or “very hard”. Finally, STIs history was elicited by asking “have you had any STIs or issues regarding your sexual and reproductive health in the last twelve months?” and answers were coded as positive if the respondent answered that yes, she had an STI in the past 12 months or that she was not sure about it.

To capture information on antibiotic adherence, answers to the question “When I had to take antibiotics or other medications, it was easy for me to remember to take the daily dose” were recoded to 1 (“yes”) if the answer to the question was “strongly agree” and recoded to 0 (“no”) if the respondent answered “agree”, “disagree”, “strongly disagree” or “don’t know”. This conservative approach was used to identify those respondents that reported the highest level of adherence to daily medications, considering that PrEP efficacy is maintained by taking it consistently on a daily basis.

To assess HIV risk perception, a potential mediator in the causal pathway for willingness to use PrEP, the question “How likely do you think it is that you will get HIV?” was measured on a three-point scale with the options “very likely,” “somewhat likely,” and “not likely” with additional options for “I already have HIV” and “don’t know”; analyses were conducted by converting it into a binary measure obtained by merging the first two option choices.

Finally, to assess the outcome of interest, willingness to use PrEP, we used the question “To what extent do you agree with the following statement: ‘If PrEP was available in my community I would probably take it’”. The response options were “strongly agree”, “agree”, “disagree”, “strongly disagree”, and “don’t know”. Because we wanted our measure to reflect as closely as possible the real anticipated intentions of participants with respect to PrEP utilization, and assuming that a moderate interest is less likely to translate into actual utilization than a strong interest, we converted the variable ‘willingness to use PrEP’ into a binary form where only those who answered ‘strongly agree’ were assigned a value of 1 (“yes”) and all others a value of 0 (“no”).

### Analytical methods

**Fig 1:**
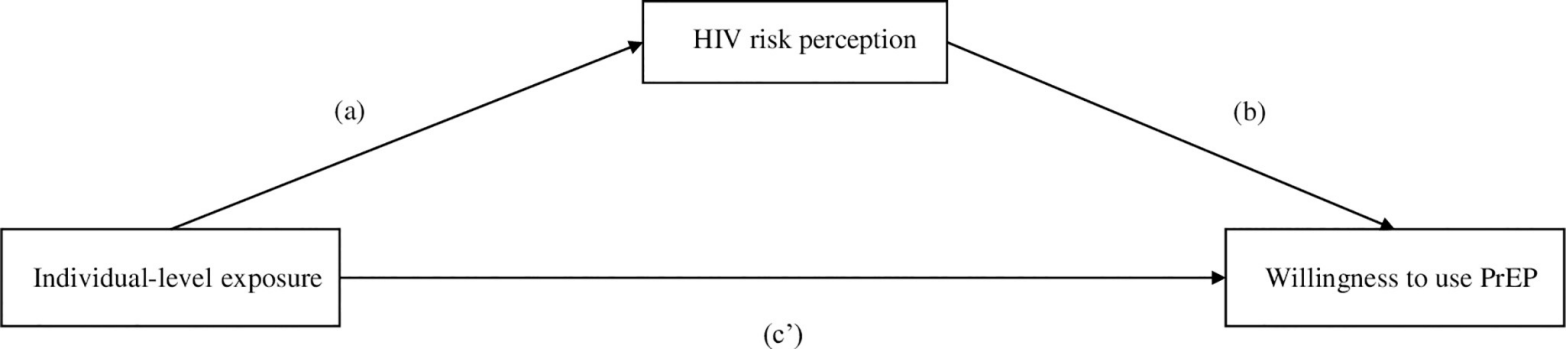
Mediation pathway of HIV risk perception and willingness to use PrEP.

First, we performed bivariate analyses with Pearson χ2 tests on categorical variables to compare participants who perceived themselves at risk of HIV to those who did not perceive to be at risk and those who expressed strong willingness to use PrEP to those who did not. Additionally, we performed a multivariate mediation analysis where HIV risk perception is the mediator and willingness to use PrEP is the final outcome of interest. To estimate the mediation model, we drew from Baron and Kenny [22] methodology: first, we ran a model with HIV risk perception as the outcome, and then we ran a model with willingness to use PrEP as the outcome, including also HIV risk perception and all the other covariates. The multivariate mediation analysis was conducted using linear probability model regressions with 500 bootstrap repetitions to construct bias-corrected confidence intervals. A simplified description of the relation under investigation is depicted in the directed acyclic graph in Fig 1: individual characteristics or covariates are individual-level exposures that may affect HIV risk self-perception (a) which in turn is hypothesized to act as a mediator and influence one’s willingness to use PrEP (b); furthermore, individual-level exposures may as well have a direct effect on willingness to use PrEP (c’). We hypothesize that, while some covariates affect willingness to use PrEP exclusively via the indirect mediated pathway, other variables may directly shift one’s willingness to use PrEP without being mediated by HIV risk perception (Fig 1). In all analyses and tables, p-value significance levels of 90% and 99% were reported together with the reference significance value of 95%.

### Ethics statement

Ethical approval for this study was obtained from the Office of Human Research Administration (OHRA) at Harvard Longwood Medical Area under IRB protocol 16–0891, the Clinton Health Access Initiative (CHAI) Scientific and Ethical Review Committee (SERC), and the Lesotho Ministry of Health Ethics and Research Committee under the protocol ID94-2016. The corresponding author was employed at CHAI during survey design and data collection phases.

## Results

Participants averaged 21.82 (± 2.98) years of age. Of all participants, 118 (39.07%) had more than one concurrent sexual partner at the time of the interview and 85 (28.14%) reported having friends who had sex for money. 113 (37.42%) reported finding it hard to negotiate condom use, 129 (42.71%) reported having friends that were beaten by male partners and 118 (39.07%) reported having been forced to have sex against their will at least once. Tertiary education institution where the interviews took place were mainly located in the university city Roma, the capital city Maseru, as well as in other districts of Lesotho (Table 1).

Overall, 57.3% of the sample perceived themselves at risk of acquiring HIV. In bivariate analyses, perceived HIV risk was strongly associated with a history of sexually transmitted infections (p<0.001), with experiences of sexual coercion (p<0.001) and with sexual partners in polygamous relationships (p = 0.003). Perceived HIV risk was also significantly associated with difficulty in negotiating condom use (p = 0.014), IPV in the network of peers (p = 0.012), inconsistent condom use (p = 0.038) and multiple concurrent sexual partnerships (p = 0.013).

Of all the respondents, 32.1% reported to be strongly willing to use PrEP if it were available in their community. Willingness to use PrEP was strongly associated with perceived HIV risk (p = 0.009), reported strong adherence to antibiotics (p = 0.001), negative attitude towards condom use (p = 0.009), IPV in the network of peers (p = 0.001) and experiences of transactional sex in the network of peers (p = 0.007). Willingness to use PrEP was also significantly associated with having sexual partners in polygamous relationships (p = 0.033) (Table 2).

**Table 2:** Bivariate analysis: HIV risk perception and willingness to use HIV PrEP by risk predictors and indicators of gender-based violence (*n* = 302).

Correlate	Perceived HIV Risk		χ^2^ (p = )	Willing to take PrEP		χ^2^ (p = )
	No	Yes		No	Yes	
	n (%)	n (%)		n (%)	n (%)	
	129 (42.7)	173 (57.3)		205 (67.9)	97 (32.1)	
Perceived HIV risk						
No	-	-		98 (76.0)	31 (24.0)	6.76 (0.009)***
Yes	-	-		107 (61.8)	66 (38.2)	
Ever been forced to have sex						
No	92 (51.1)	88 (48.9)	13.41 (<0.001)***	126 (70.0)	54 (30.0)	1.02 (0.312)
Yes	35 (29.7)	83 (70.3)		76 (64.4)	42 (35.6)	
Have friends who were beaten by male partner						
No	83 (48.5)	88 (51.5)	6.27 (0.012)**	130 (76.0)	41 (24.0)	11.76 (0.001)***
Yes	44 (34.1)	85 (65.9)		74 (57.4)	55 (42.6)	
Have friends who have had sex for money						
No	95 (45.0)	116 (55.0)	2.96 (0.085)*	153 (72.5)	58 (27.5)	7.15 (0.007)***
Yes	29 (34.1)	56 (65.9)		48 (56.5)	37 (43.5)	
Find it hard to negotiate condom use						
No	91 (48.2)	98 (51.8)	6.09 (0.014)**	131 (69.3)	58 (30.7)	0.47 (0.491)
Yes	38 (33.6)	75 (66.4)		74 (65.5)	39 (34.5)	
Inconsistent condom use						
No	77 (38.5)	123 (61.5)	4.29 (0.038)**	132 (66.0)	68 (34.0)	0.96 (0.327)
Yes	52 (51.0)	50 (49.0)		73 (71.6)	29 (28.4)	
Report to dislike condoms						
No	107 (43.3)	140 (56.7)	0.48 (0.488)	175 (70.8)	72 (29.2)	6.75 (0.009)***
Yes	19 (38.0)	31 (62.0)		26 (52.0)	24 (48.0)	
Report strong adherence to antibiotics						
No	115 (44.2)	145 (55.8)	1.75 (0.185)	186 (71.5)	74 (28.5)	11.47 (0.001)***
Yes	14 (33.3)	28 (66.7)		19 (45.3)	23 (54.7)	
More than one current sexual partner						
No	89 (48.4)	95 (51.6)	6.15 (0.013)**	121 (65.8)	63 (34.2)	0.97 (0.325)
Yes	40 (33.9)	78 (66.1)		84 (71.2)	34 (28.8)	
Partner has multiple partners or unsure						
No	65 (52.8)	58 (47.2)	8.70 (0.003)***	92 (74.8)	31 (25.2)	4.55 (0.033)**
Yes	64 (35.7)	115 (64.3)		113 (63.1)	66 (36.9)	
Had STIs in past 12 months or unsure						
No	111 (48.5)	118 (51.5)	14.42 (<0.001)***	155 (67.7)	74 (32.3)	0.01 (0.936)
Yes	15 (22.4)	52 (77.6)		45 (67.2)	22 (32.8)	

*** p < 0.01

** p < 0.05

* p < 0.1

Observed n ranged from 296 to 302 due to item-specific missing values.

A multivariate mediation analysis was conducted using the same covariates included in the bivariate analyses, and controlling also for age and institution. Table 3 presents the results of each part of the mediation pathway separately, by showing the indirect effects of the covariates on the mediator (a) and the effect of the mediator on the outcome (b) and jointly by reporting the bias-corrected bootstrap confidence interval; as hypothesized, perceived HIV risk was found to be a significant mediator, being associated with 11.5 percentage points increase in likelihood of using PrEP (p = 0.041). Three covariates showed a significant mediated indirect pathway for willingness to use PrEP. Multiple concurrent sexual partnership was associated with 16.1 percentage points increase in likelihood of self-perceived HIV risk (p = 0.007), while having sexual partners in polygamous relationships was associated with 17.8 percentage points increase in likelihood of self-perceived HIV risk (p = 0.002) and the mediated indirect effect accounted for 18.2% of its total effect; similarly, history of sexually transmitted infections was also found to be statistically significant (p = 0.009) and associated with 16.5 percentage points increase in likelihood of self-perceived HIV risk. Having experienced sexual coercion only approached significance at 95% confidence level (p = 0.059).

**Table 3:** Indirect effects and total effects for multivariate mediation analysis of willingness to use PrEP (*n* = 282).

	Indirect Effect					Direct Effect		Total Effect	
	Effect Size				BC Bootstrap CI	Effect Size		Effect Size	BC Bootstrap CI
	(a)	(p = )	(b)	(p = )		(c’)	(p = )	(c) = (a)*(b)+(c’)	
	→ Perceived HIV risk		→ Willing to use PrEP			Willing to use PrEP		Willing to use PrEP	
			0.1154**	0.041					
Ever been forced to have sex	0.1103*	0.082			-0.0006–0.0410	-0.0158	0.790	-0.0031	-0.1190–0.1086
Have friends who were beaten by male partner	0.0805	0.168			-0.0008–0.0352	0.1499***	0.009	0.1592***	0.0345–0.2707
Have friends who have had sex for money	-0.0002	0.996			-0.0192–0.0142	0.0901	0.144	0.0901	-0.0342–0.2165
Find it hard to negotiate condom use	0.0707	0.217			-0.0024–0.0328	0.0049	0.931	0.0131	-0.1093–0.1191
Inconsistent condom use	-0.0542	0.384			-0.0281–0.0066	0.0235	0.693	0.0173	-0.1123–0.1324
Report to dislike condoms	0.0244	0.740			-0.0126–0.0284	0.1916**	0.010	0.1944**	0.0594–0.3678
Report strong adherence to antibiotics	0.0150	0.851			-0.0162–0.0294	0.2308***	0.004	0.2326***	0.0514–0.3793
More than one current sexual partner	0.1614***	0.007			0.0022–0.0512	-0.0547	0.347	-0.0362	-0.1513–0.0781
Partner has multiple partners or unsure	0.1776***	0.002			0.0723–0.2970	0.0921	0.114	0.1125*	-0.0163–0.2253
Had STIs in past 12 months or unsure	0.1655***	0.009			0.0013–0.0544	-0.0843	0.202	-0.0653	-0.2062–0.0575

*n* = 282 (variations across analyses due to missing data on covariates and mediators variables). BC Bootstrap CI = bias-corrected bootstrap confidence intervals.

This analysis reports the effects of the compound path from the variables on the left-hand side to the outcome variable Willingness to use PrEP through the mediator Perceived HIV risk.

*** p<0.01,

** p<0.05,

* p<0.1

As expected, some covariates were directly associated with willingness to use PrEP and their effect was not mediated by HIV risk perception: those who reported strong adherence to antibiotics were 23.1 percentage points more likely to express willingness to use PrEP than those who did not (p = 0.004), and those who reported to dislike condoms were 19.1 percentage points more likely to be willing to use PrEP than those who did not report aversion to condom use. Interestingly, IPV in the network of peers was also directly associated with willingness to use PrEP and its effect was not significantly mediated by HIV risk perception: those who had friends who experienced intimate partner violence were 14.9 percentage points more likely to be willing to use PrEP than those who did not report IPV in their network of peers (p = 0.009) (Table 3).

## Discussion

This study on willingness to use PrEP among female university students in Lesotho was prompted by the introduction, in April 2016, of new ministerial guidelines on the use of antiretroviral therapy for HIV prevention and treatment, which introduced oral PrEP as a new HIV prevention service for high-risk individuals in Lesotho. The study focused on young women because they had been previously identified as one of the populations with the highest HIV incidence in the country, and because they may experience intimate partner violence, engage in transactional sex, and have multiple concurrent sexual partnerships, which have been previously associated with increased likelihood of HIV infection. In particular, the study focused on women attending tertiary education institutions in Lesotho at the time of the survey.

In this sample of 302 female university students, more than half (57.3%) considered themselves very likely or somewhat likely to acquire HIV, and one in three (32.1%) reported strong intentions to use PrEP if it were available in their community. A multivariate mediation analysis indicated that multiple concurrent sexual partnerships and having a partner in polygamous relationships had independent associations with perceived HIV risk, and perceived HIV risk significantly mediated their effect on willingness to use PrEP. As expected, then, HIV risk perception did not mediate the effect of reporting strong adherence to antibiotics and reporting negative attitudes towards condom use (i.e. agree or strongly agree with the statement “I don’t like condoms”) on willingness to use PrEP: these two factors affect the likelihood of PrEP uptake independently from perceived HIV risk. Importantly, IPV in the network of peers was strongly associated with increased willingness to use PrEP and HIV risk perception did not significantly mediated this effect. This seems to confirm that IPV in peer networks may relate to one’s individual risk perception through direct involvement of peers in shaping couple power dynamics, as previously suggested, or because similar baseline characteristics act as co-factors in determining relationships and forming networks: in both cases, these hypotheses support the conclusion that findings that relate to women who experience IPV in their network of peers may as well apply to women who directly experience IPV. Indirectly, these findings also suggest that women who are part of networks where IPV is common feel more at risk of acquiring HIV [23–25], although they may not consider such risk to be necessarily imminent or current. Another possible explanation to the fact that IPV in the network of peers had a direct effect on willingness to use PrEP, not mediated by HIV risk perception, is that women who witness IPV in their peers’ network may not feel directly at risk of contracting HIV but would nevertheless prefer to be protected, perhaps because their exposure to IPV dynamics has made them more aware and therefore less prone to risk taking.

The findings of this study have significant implications for HIV prevention programs in Lesotho. First, they strongly suggest that MoH PrEP guidelines be reviewed to include women who experience IPV among the key populations to whom PrEP should be targeted: this would also ensure consistency between PrEP guidelines and HIV testing guidelines, where this key population is identified as one among those at higher risk; the results of this study seem to support the use of indirect questioning to elicit information about IPV and suggest that IPV in their network of peers may actually translate into increased will for HIV prevention. Second, women in multiple concurrent sexual partnerships perceive themselves at higher risk of acquiring HIV, which supports the inclusion of individuals with multiple concurrent sexual partners among the categories to whom PrEP service provision should be targeted based on the 2016 ministerial guidelines. Nevertheless, an objective assessment of individuals’ risk behavior is always recommended, because an individual’s self-perception of HIV risk may not coincide with one’s objective risk of HIV. Third, there exist predictors of PrEP uptake that do not depend on HIV risk perception, and these include adherence to daily medications and negative attitudes towards condoms: women who expressed a negative attitude towards condom use are significantly more willing to use PrEP than their peers, suggesting that this subpopulation may be likely to use PrEP in lieu of condoms regardless of their perceived risk of contracting HIV: based on these findings, condom use should be particularly encouraged and monitored while scaling up PrEP in similar populations that present high degree of aversion to condom use. Finally, strong self-reported adherence to daily antibiotics is a significant independent predictor of anticipated PrEP uptake, confirming adherence as one of the possible barriers to PrEP utilization [26].

HIV risk perception is a significant predictor of willingness to use PrEP; however, similarly to previous studies [27], willingness to use PrEP is not always driven by higher HIV risk self-perception, since several women in the sample who did not feel at particular risk of contracting HIV showed interest in this preventive measure: this apparent contradiction may be explained by higher risk aversion as well as by the fact that certain individual characteristics, such as adversity to condom use, constitute by themselves strong predictors of PrEP uptake, regardless of current HIV risk self-perception. Future campaigns to expand PrEP utilization should therefore focus as well on factors that are not necessarily linked to one’s HIV risk self-perception.

This study had several limitations. One limitation is that the survey was only conducted with university students, which is a subgroup that may not be generalizable to the larger population of adolescent girls and young women in Lesotho. Furthermore, the participants were concentrated in the capital city Maseru and the university city Roma, which limits the extent to which one can extrapolate to more rural populations. However, since the HIV burden is highest in the urban center of Maseru, focusing on this population does make sense in developing an HIV prevention strategy for the country. Yet, more research on less educated and more rural populations may be warranted in the development of a comprehensive HIV prevention and PrEP strategy for adolescent girls and young women in Lesotho. One other limitation of the study is that the data is all self-reported. Especially since the study is dealing with sensitive issues regarding sexual behavior, respondents may have felt a social pressure to answer questions in a certain way. While attention was taken in the wording of questions as to not bias participants and participants were able to complete the survey in private, there remains a risk of social desirability bias, or respondents not understanding the questions. It also must be acknowledged that we were asking questions about future behavior, which is very difficult to do given that participants have a hard time projecting attitudes and behavior to the future.

This study is the first of its kind in Lesotho to examine HIV risk perception and its relation to HIV risk factors, IPV predictors, and their association with anticipated uptake of PrEP among female university students. Follow-up research should be conducted to examine actual utilization of PrEP among female university students in Lesotho and understand whether this aligns with anticipated intentions of use.

## Supporting information

S1 Data (XLS)

S2 Data (XLS)

S3 Data (XLS)

S4 Data (XLS)

S5 Data (XLS)

S6 Data (XLS)

S7 Data (XLS)

S8 Data (XLS)

S9 Data (XLS)

S10 Data (XLS)

S1 Table (XLS)

S2 Table (XLS)
